# Spironolactone alleviates schizophrenia-related reversal learning in Tcf4 transgenic mice subjected to social defeat

**DOI:** 10.1038/s41537-022-00290-4

**Published:** 2022-09-29

**Authors:** Marius Stephan, Jonathan Schoeller, Florian J. Raabe, Andrea Schmitt, Alkomiet Hasan, Peter Falkai, Niels Jensen, Moritz J. Rossner

**Affiliations:** 1 Department of Psychiatry and Psychotherapy, Laboratory of Molecular Neurobiology, University Hospital, LMU Munich, Munich, Germany; 2 International Max Planck Research School for Translational Psychiatry (IMPRS-TP), Munich, Germany; 3 Laboratory of Neuroscience (LIM27), Institute of Psychiatry, University of São Paulo, São Paulo, Brazil; 4 Department of Psychiatry, Psychotherapy and Psychosomatics of the University Augsburg, University of Augsburg, Medical Faculty, Augsburg, Germany; 5 Max Planck Institute of Psychiatry, Munich, Germany

**Keywords:** Schizophrenia, Schizophrenia

## Abstract

Cognitive deficits are a hallmark of schizophrenia, for which no convincing pharmacological treatment option is currently available. Here, we tested spironolactone as a repurposed compound in Tcf4 transgenic mice subjected to psychosocial stress. In this ‘2-hit’ gene by environment mouse (GxE) model, the animals showed schizophrenia-related cognitive deficits. We had previously shown that spironolactone ameliorates working memory deficits and hyperactivity in a mouse model of cortical excitatory/inhibitory (E/I) dysbalance caused by an overactive NRG1-ERBB4 signaling pathway. In an add-on clinical study design, we used spironolactone as adjuvant medication to the standard antipsychotic drug aripiprazole. We characterized the compound effects using our previously established Platform for Systematic Semi-Automated Behavioral and Cognitive Profiling (PsyCoP). PsyCoP is a widely applicable analysis pipeline based on the Research Domain Criteria (RDoC) framework aiming at facilitating translation into the clinic. In addition, we use dimensional reduction to analyze and visualize overall treatment effect profiles. We found that spironolactone and aripiprazole improve deficits of several cognitive domains in Tcf4tg x SD mice but partially interfere with each other’s effect in the combination therapy. A similar interaction was detected for the modulation of novelty-induced activity. In addition to its strong activity-dampening effects, we found an increase in negative valence measures as a side effect of aripiprazole treatment in mice. We suggest that repurposed drug candidates should first be tested in an adequate preclinical setting before initiating clinical trials. In addition, a more specific and effective NRG1-ERBB4 pathway inhibitor or more potent E/I balancing drug might enhance the ameliorating effect on cognition even further.

## Introduction

Most psychiatric disorders, such as autism spectrum disorder, major depression, and schizophrenia, arise from complex interactions of genetic and environmental influences^1–3^. Mouse models that use a combination of genetic alterations with a mild influence on neurodevelopment as the first ‘hit’ and an environmental stressor as the second ‘hit’ are thought to yield psychiatric disease models with improved face and predictive validity over simple genetic and pharmacological models^4^.

The basis of such a ‘2-hit’ model is typically a moderate loss or gain of function genetic mouse line harboring a genetic risk factor previously identified in genome-wide association studies (GWAS) in humans. In this study, we focus on Transcription factor 4 (*TCF4*), a gene strongly associated not only with the risk of developing schizophrenia but also bipolar disorder and major depressive disorder^5,6^. Mice moderately overexpressing *Tcf4* (*Tcf4*tg) or with a depletion of the long variants of the TCF4 protein (*Tcf4*Ex4δ+/−) were both shown to display cognitive impairments, suggesting an inverted U-shape relationship of cognitive function and *Tcf4* gene dosage. This is a phenomenon commonly found in neurobiological systems, including mental disorders such as schizophrenia spectrum disorders^7–9^. Moreover, TCF4 has recently been identified as one of the strongest genetic risk genes that is upregulated in upper cortical layer neurons by scRNAseq analysis from human prefrontal cortex samples^10^ and has been previously predicted as a “master regulator” of schizophrenia gene networks^11^. Moreover, TCF4 genotype has been linked to cognition and gating, including a human 2-hit model^12,13^.

The environmental factor, on the other hand, can be quite diverse, with internal and external influences acting at different timepoints and on different systems, to produce a similar clinical phenotype. For example, the risk for developing schizophrenia can already be increased in utero by an inflammatory response of the mother’s immune system to a viral infection. It can also occur as late as the age of onset, in the form of single stressful events and extended stress exposure^14,15^. Chronic psychosocial stress can be modeled in mice in the resident intruder paradigm during adolescence. In this setup, a male test mouse (intruder) is exposed to a territorially aggressive mouse (resident) in its home cage. This leads to a variety of psychiatric endophenotypes including cognitive dysfunction^16^.

In a previous study, we established a standardized platform for systematic cognitive and behavioral profiling (PsyCoP) to characterize *Tcf4* transgenic mice (*Tcf4*tg) subjected to social defeat (SD) during adolescence as a ‘2-hit’ gene by environment interaction (GxE), schizophrenia-related mouse model^17^. The PsyCoP test battery consists of a diverse panel of well-established behavioral tests and most of the procedures are at least partially automated to minimize the influence of the experimenter. In this and previous studies^17,18^, we found impairments in cognitive flexibility and fear memory in the *Tcf4*tg x SD (*Tcf4*SD) GxE mouse model, making it particularly suited to study aspects of cognitive symptoms of schizophrenia spectrum disorders in pharmacological validation experiments. Although the genetics of psychiatric disorder endophenotypes were found to be complex, modeling selected endophenotypes based on conserved neurobiological systems is thought to yield higher construct validity than trying to model complex neuropsychiatric disorders in mice^19,20^.

For our automated analysis pipeline, we categorized the single variables according to the behavioral traits and domains defined in the Research Domain Criteria (RDoC) framework and used dimension-reduction analysis for visualization of the overall behavioral and cognitive profile. The RDoC framework is based on the neurobiological basis of conserved traits^21^ and neurobiological structures and associated phenotypes are categorized on multiple levels into 6 top-level domains: cognitive systems, sensorimotor systems, positive valence systems, negative valence systems, arousal, and regulatory systems, as well as social processes^22^. In contrast to the symptom-based categorization commonly used in clinical practice, these domains may represent at least partially distinct biological systems.

One such translational preclinical study is the validation of spironolactone as a compound to modulate the excitatory/inhibitory (E/I) balance in the cortex by inhibition of the ERBB4 receptor^23^. NRG1 is a ligand of the ERBB4 receptor and overexpression of NRG1 Type I in *Nrg1*tg mice leads to overactivity of the NRG1-ERBB4 signaling pathway^24^. Altered NRG1-ERBB4 signaling was found to be associated with schizophrenia risk and impairment of cognitive traits in patients, but also healthy controls^25–27^. A defined set of cognitive and behavioral traits, such as working memory span in the Y-maze test, was used to test spironolactone’s effect on *Nrg1* overexpressing (*Nrg1*tg) mice, as these mutant mice display schizophrenia-like cognitive endophenotypes^28^. Chronic spironolactone treatment was found to ameliorate this effect, validating its value as a candidate compound for the treatment of cognitive symptoms in schizophrenia patients^23^.

This finding is worth investigating further, as there is still a clinical need for compounds that are effective against negative and cognitive symptoms of schizophrenia as current medications mostly affect only positive symptoms^29,30^. However, pharmaceutic companies have to critically evaluate their investments in the field of psychotic disorders, raising the importance of psychosocial interventions, but also academic research in the field of psychopharmacological drug development^31^. In fact, drug repurposing could be an additional strategy for the development of new treatments for schizophrenia and other mental disorders^32,33^. Spironolactone, for example, is an FDA and EMA approved compound that was first identified as a candidate substance in cell-based drug repurposing screening assays in academic research^23^. Based on these considerations and the promising results in the *Nrg1*tg mouse model, a clinical trial is currently underway, testing spironolactone as adjuvant medication patients treated with antipsychotics^34^.

Although *Nrg1*tg mice are a valuable genetic tool regarding E/I dysbalance, it was not paired with an environmental factor, and spironolactone was selected for its action specifically on the signaling networks deregulated in the *Nrg1*tg mouse model, raising the question of its efficacy and if other disease-relevant systems are affected as well. Moreover, neither *NRG1* nor *ERBB4* are validated genetic risk genes from GWAS studies. To improve the face and etiological validity, it would be prudent to test the spironolactone in an orthogonal ‘2-hit’ mouse model displaying cognitive deficits.

In this study, we report the results of such a combinatorial treatment study with aripiprazole and spironolactone in the 2-hit *Tcf4*tg x social defeat (*Tcf4*SD) mouse model. We found improvements of some cognitive aspects in mice treated with spironolactone alone. Interestingly, co-treatment with aripiprazole interfered with this effect, suggesting a detrimental interaction of both compounds. This finding highlights the importance of testing compounds in combination already on the level of preclinical translational studies, to reveal treatment interactions prior to clinical studies.

## Results

**Fig. 1:**
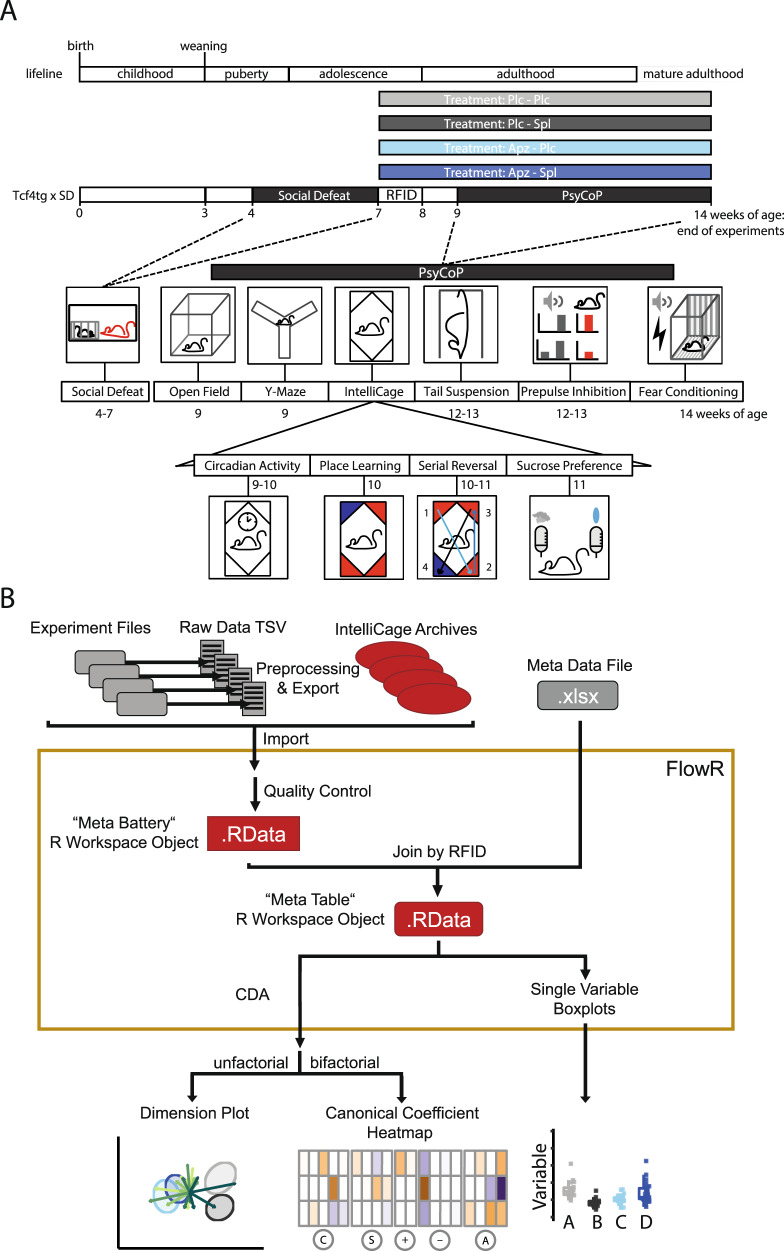
Experimental layout and data flow. **A** Experimental timeline. *Tcf4*tg-mice were all subjected to social stress during early adolescence (*Tcf4*tgSD). The behavioral tests were performed according to the previously published PsyCoP protocol with tail suspension test and pre-pulse inhibition test reversed and in order of increasing aversiveness^17^. **B** Overview of the data analysis pipeline. Data acquisition and preprocessing were semi-automated. All data were collected in a RData dump for further analysis in FlowR. The variables were categorized in domains following the Research Domain Criteria (RDoC) framework. Canonical discriminant analysis (CDA) was used for dimension reduction. The resulting neurocognitive profiles were visualized in dimension plots of the dimension reduction and a heatmap of the weights of each variable in the CDA result.

In this study, we used the previously established profiling platform PsyCoP to assess the effect of spironolactone, particularly on the cognitive performance in the *Tcf4*SD ‘2-hit’ mouse model^17^. We treated the mice either with aripiprazole alone or in combination with spironolactone as an add-on treatment (Fig. 1A). We chose aripiprazole, because, in contrast to other commonly prescribed antipsychotics such as quetiapine, olanzapine, or risperidone, it acts as a partial agonist on the D2R and its functional selectivity has been hypothesized to positively influence hypodopaminergic states in the prefrontal cortex and thus cognition^35^. No healthy control groups were included as both compounds used in this study are well known and the focus of this study is on their effect on the reduced cognitive performance of the *Tcf4*tg x SD (*Tcf4*SD) mouse model. Four chronic treatment groups were tested: A double placebo reference group (Plc-Plc), an aripiprazole-only group (Apz-Plc), a spironolactone-only group (Plc-Spl), and a group treated with a combination of both compounds (Apz-Spl).

We did not observe reduced weight or water consumption in treated mice (Suppl. Fig. 1) and did not detect major deviations from the normal distribution (Suppl. Fig. 2). The whole dataset was analyzed by categorization in the corresponding RDoC domains, visualization of Z-scores of the main effects, and dimension reduction in a canonical discriminant analysis (Fig. 1B).

### Cognitive systems—aripiprazole treatment interferes with spironolactone’s ameliorating effect on reversal learning in *Tcf4*SD mice

**Fig. 2:**
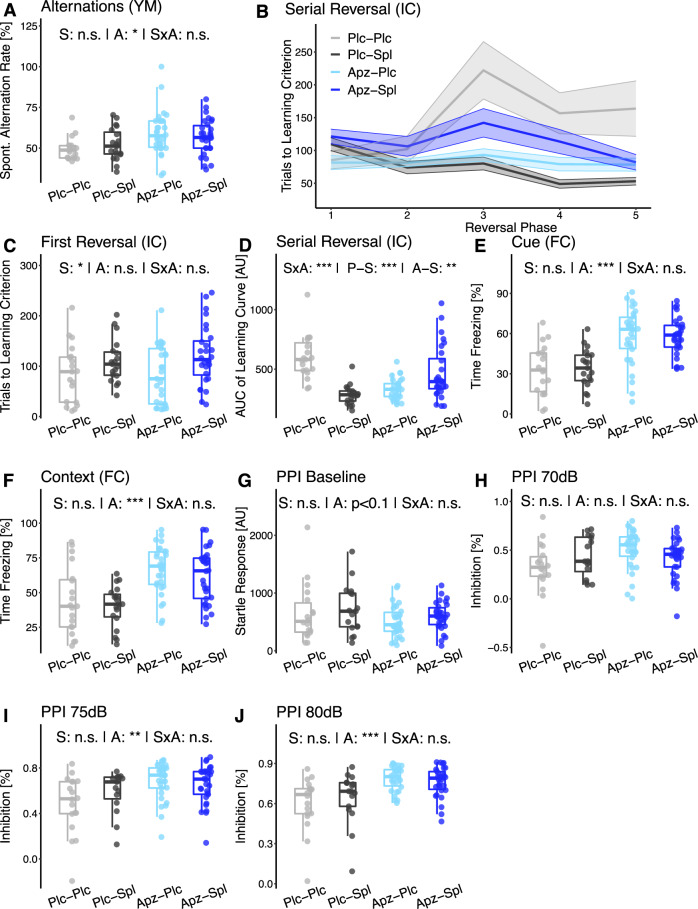
Adjuvant spironolactone treatment interferes with the beneficial effect of aripiprazole on serial reversal learning performance. Variables measuring aspects of the cognition domain are shown in panels (**A**–**F**). **A** The rate of spontaneous alternations in the Y-maze test (YM) was used as an indicator of working memory performance. **B** The number of trials a subject needs to reach the learning criterion in the sequential probability ratio test (SPRT) reflects the learning speed, with lower values indicated higher learning performance. The graph shows group means of that learning speed with the standard error of the mean (SEM). **C** The performance in the first reversal indicates learning flexibility following spatial learning in the place learning phase. **D** Performance in the more demanding serial reversal learning task was quantified as area under the learning curve (AUC), computed as sum of the rolling mean across all reversal phases. **E**, **F** Freezing behavior in the cued (**E**) and contextual (**F**) fear conditioning tasks. **G**–**J** Prepulse inhibition results. Included are (**G**) baseline startle and prepulse inhibition at sound levels of **H** 70 dBA, **I** 75 dBA, and **J** 80 dBA. Data are shown as box plots with whiskers extending to no more than 1.5-fold IQR; *p < 0.05, **p < 0.01, ***p < 0.001, n.s. not significant; *p*-values are FDR-adjusted and refer to Wilk’s lambda testing two-way ANOVA; *n* = 19/19/30/30; Plc placebo, Spl spironolactone treatment, Apz aripiprazole treatment, S spironolactone term, A aripiprazole term, SxA interaction term, P-S spironolactone effect in placebo-treated mice, A-S spironolactone effect in aripiprazole-treated mice.

One of the cognitive traits modulated by spironolactone in the previous study with *Nrg1* transgenic mice was working memory performance in the Y-maze test^23^. We did not observe a similar increase in alternation rate in the current study, which might indicate no impact on working memory performance in response to chronic spironolactone treatment (S) in *Tcf4*SD mice. Instead, we found that aripiprazole (A) increased the spontaneous alternation rate (Fig. 2A; S: F(1, 85) = 0.108, *p* = 0.901; A: F(1, 85) = 9.51, *p* = 0.0105; SxA: F(1, 85) = 1.52, *p* = 0.354). Notably, in the previous behavioral profiling, *Tcf4*SD mice displayed no reduction in alternation rate in the Y-maze test compared to non-stressed wildtypes, suggesting no working memory impairment^17^.

In the first reversal phase of the IntelliCage spatial learning task (Fig. 2B–D), spironolactone-treated mice performed slightly worse, with a mean number of trials needed to reach the learning criterion of 109.6 ± 9.8 and 121.4 ± 11.1 for Plc-Spl and Apz-Spl, respectively, compared to 85.2 ± 13.3 and 81.5 ± 10.6 for Plc-Plc and Apz-Plc (Fig. 2C; S: F(1, 85) = 8.48, *p* = 0.0163). We next analyzed the mean success rate of the second day of the place learning and the first day of reversal to show the drop in performance upon corner change (Suppl. Fig. 3A, B). In contrast to the first reversal, when looking at the following phases, the strongest improvement was in the spironolactone-only group (Plc-Spl) (Fig. 2B, D). Overall, spironolactone and aripiprazole were interacting in the serial reversal learning task (Fig. 2D; SxA: F(1, 85) = 46.5, *p* = 1.80 × 10^−8^). Plc-Spl mice were significantly faster compared to Plc-Plc (P-S: F(1, 36) = 46.7, *p* = 5.39 × 10^−8^), while Apz-Spl were significantly slower than Apz-Plc mice (A-S: F(1, 55) = 8.23, *p* = 5.84 × 10^−3^) (Fig. 2B, D).

This is also reflected in a similar interaction of spironolactone and aripiprazole treatment on the mean success rate (SxA: F(1, 94) = 5.43, *p* = 0.0256), where spironolactone significantly improves success rate alone, but not in combination with aripiprazole (P-S: F(1, 36) = 11.8, *p* = 1.48 × 10^−3^; A-S: F(1, 58) = 0.787, *p* = 0.379) (Suppl. Fig. 3C, D). The rewarded trial rate was reduced in Apz-Spl treated mice only, but not in Apz-Plc and Plc-Spl treatment groups (SxA: F(1, 74) = 14.8, *p* = 2.45 × 10^−4^; P-S: F(1, 31) = 2.55, *p* = 0.120; A-S: F(1, 46) = 16.6, *p* = 1.82 × 10^−4^) (Suppl. Fig. 3E). However, the difference between Apz-Spl treated mice and the other groups decreased until it was absent in reversal phase 5 (Suppl. Fig. 3F).

**Fig. 3:**
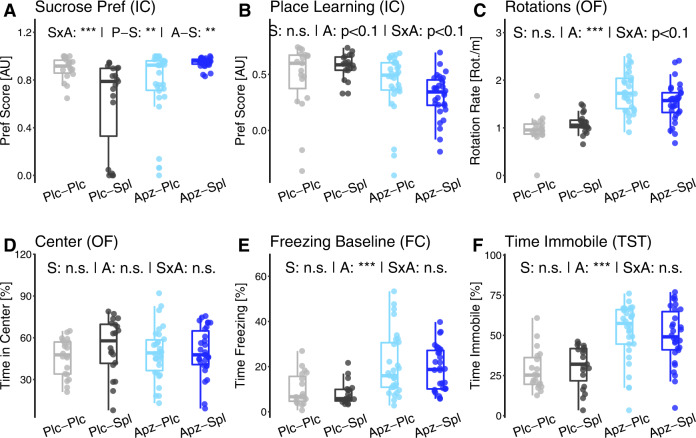
Aripiprazole treatment increases measures of negative valence in *Tcf4*SD mice. All three variables associated with the positive valence domain. **A** Sucrose preference quantified in an IntelliCage task, measured as preference score from −1 (absolute avoidance) to 1 (exclusive preference). **B** The same preference score representing preference for the rewarded corner in a spatial positive reinforcement-learning paradigm in the IntelliCage (place learning). **C** Path choice quantified as rotation rate in the open field test was interpreted as nervous or hectic behavior. **D** Center time in the open field test and **E** baseline freezing behavior in the fear conditioning test were used as proxies of anxiety level. **F** Time immobile in the tail suspension test quantifying the absence of struggling behavior. Data are shown as box plots with whiskers extending to no more than 1.5-fold IQR; *p < 0.05, **p < 0.01, ***p < 0.001, n.s. not significant; *p*-values are FDR-adjusted and refer to Wilk’s lambda testing two-way ANOVA; *n* = 19/19/30/30; Plc placebo, Sp spironolactone treatment, Apz aripiprazole treatment, S spironolactone term, A aripiprazole term, SxA interaction term, P-S spironolactone effect in placebo-treated mice, A-S spironolactone effect in aripiprazole-treated mice.

Spironolactone treatment had no significant effect on freezing behavior in both cued (Fig. 2E) and contextual (Fig. 2F) fear memory tasks, whereas aripiprazole-treated mice showed an increase in time spent freezing in both tasks (A: Cue F(1, 85) = 36.7, *p* = 3.49 × 10^−7^; F(1, 85) = 24.6, *p* = 2.28 × 10^−5^). Of note, the baseline freezing time was increased in aripiprazole-treated mice, which indicates that the corresponding effects in the fear conditioning task cannot be attributed unequivocally to enhanced memory performance (Fig. 3E).

### Sensorimotor systems—aripiprazole enhances prepulse inhibition

*Tcf4*tg mice were shown to suffer from sensorimotor gating deficits in a prepulse inhibition (PPI) test, while social defeat as a ‘late-stage’ environmental factor had no influence^17^. Although the underlying mechanism of the dysfunction is still largely unknown, it is thought to reflect the gating impairments characteristic for schizophrenic patients^36^.

In treated *Tcf4*SD mice, we found that aripiprazole significantly ameliorated the PPI deficit for prepulse levels of 75 dBA and 80 dBA, whereas spironolactone treatment had no impact (Fig. 2H–J; PPI 75 dB: A: F(1, 85) = 11.1, *p* = 5.23 × 10^−3^; S: F(1, 85) = 0.426, *p* = 0.715; SxA: F(1, 85) = 1.50, *p* = 0.354; PPI 80 dB A: F(1, 85) = 19.1, *p* = 1.79 × 10^−4^; S: F(1, 85) = 0.404, *p* = 0.715; SxA: F(1, 85) = 3.30, *p* = 0.160). Notably, the baseline startle amplitude was not significantly changed (PPI Baseline A: F(1, 85) = 5.14, *p* = 0.0704, S: F(1, 85) = 1.72, *p* = 0.339, SxA: F(1, 85) = 0.006, *p* = 1.00), suggesting that these effects were not the consequences of a potential sedative effect of aripiprazole (Fig. 2G).

### Positive and negative valence systems—aripiprazole treatment increases measures of negative valence in *Tcf4*SD mice as a side effect

Positive and negative valence systems were not the focus of this treatment study. However, potential side effects, for example, effects on motivation and positive reinforcement learning can be detected when a full behavioral profile is acquired. Thus, we think that it is important to characterize the effects of potential clinical compounds on all relevant cognitive and behavioral domains, when searching for novel compounds or compound combinations, even when focusing on a specific set of endophenotypes.

In the previously published behavioral characterization of *Tcf4*SD mice, measures of positive valence systems were only influenced by social defeat, with no impact of *Tcf4* gene dosage^17^.

In this treatment study, we did observe an interaction of spironolactone and aripiprazole treatment in sucrose preference (Fig. 3A; SxA: F(1, 85) = 26.2, *p* = 1.34 × 10^−5^). Treatment with spironolactone and aripiprazole alone decreased the preference of the mice for sucrose water (P-S: F(1,35) = 9.78, *p* = 3.54 × 10^−3^, A-S: F(1,58) = 11.0, *p* = 1.61 × 10^−3^), while a combination of both brought preference back to the level of the Plc-Plc group. Looking at the distribution of these effects, however, the group differences appear to be driven by subpopulations, suggesting that only a subgroup of mice might experience this effect for unknown reasons. A similar effect was observed in the characterization of the *Tcf4*SD mouse model^17^.

In the place preference test for positive reinforcement learning, the preference did not differ significantly between treatment groups (Fig. 3B). These results suggest a similar interaction observed in the serial reversal learning task, but the effect did not survive FDR correction (SxA: *p* (unadjusted) = 0.0251, *p* (adjusted) = 0.0704).

The rotation rate (Fig. 3C), however, was significantly increased in response to aripiprazole treatment (A: F(1, 85) = 52.3, *p* = 3.69 × 10^−9^), with no significant influence of spironolactone (S: *p* = 0.286). In the open field test, the time spent in the center area of the arena did not differ between groups (Fig. 3D). However, baseline freezing behavior in a novel environment was significantly higher in aripiprazole-treated mice compared to the corresponding placebo groups (Fig. 3E; A: F(1, 85) = 37.7, *p* = 1.97 × 10^−4^). As for all activity-dependent measures, this is likely to be a consequence of the decrease in locomotor activity caused by aripiprazole (see below) and likely not reflecting increased anxiety. A similar effect was observed in the tail suspension test, where the time spent immobile was significantly higher for aripiprazole-treated mice compared to placebo, without influence of spironolactone treatment (Fig. 3F; A: F(1, 85) = 18.7, *p* = 2.95 × 10^−7^).

### Arousal and regulatory systems—aripiprazole treatment reduces locomotor activity and dampens circadian activity amplitude

Arousal and regulatory systems in the RDoC system include general activity and circadian regulation. *Tcf*4SD mice were shown to be hyperactive in the open field test in the PsyCoP phenotyping study, with an interaction of genetic predisposition and environmental hit^17^.

**Fig. 4:**
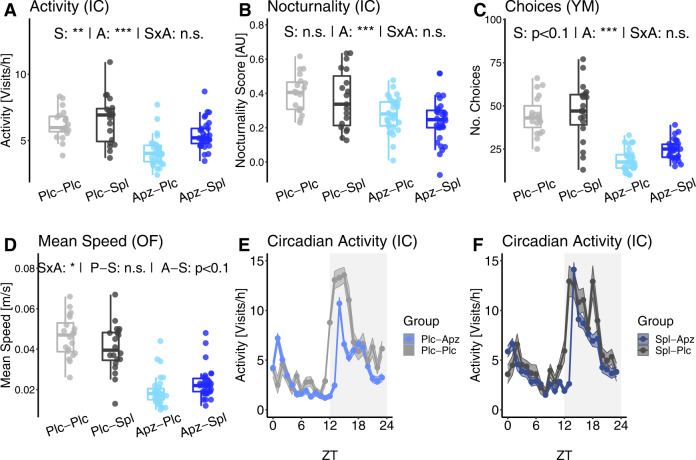
Aripiprazole reduces measures of arousal in *Tcf4*SD mice. **A** General locomotor activity level in the IntelliCage (IC) was quantified as instantaneous visit frequency. **B** In addition, the nocturnality of activity in the IC was measured with a preference (nocturnality) score similar to sucrose preference, where −1 indicates exclusive daytime activity and +1 only nighttime activity. **C**, **D** Moreover, spontaneous, novelty-induced activity was assessed as (C) total number of choices in the Y-maze test (YM) and (D) mean speed in the open field test (OF). **E**, **F** Circadian activity distribution in 1-h bins, quantified as instantaneous frequency. The data is shown as group (line) and its standard error (ribbon). Data are shown as box plots with whiskers extending to no more than 1.5-fold IQR; *p < 0.05, **p < 0.01, ***p < 0.001, n.s. not significant; *p*-values are FDR-adjusted and refer to Wilk’s lambda testing two-way ANOVA; *n* = 19/19/30/30; Plc placebo, Spl spironolactone treatment, Apz aripiprazole treatment, S spironolactone term, A aripiprazole term, SxA interaction term, P-S spironolactone effect in placebo-treated mice, A-S spironolactone effect in aripiprazole-treated mice.

Here, aripiprazole-treated mice displayed reduced locomotor activity in all four measures of activity and arousal, ameliorating the novelty-induced hyperactivity of our disease model (Fig. 4A–D). General activity in the IntelliCage was lower compared to non-aripiprazole-treated mice, while spironolactone treatment increased the activity level (Fig. 4A; S: F(1, 85) = 11.2, *p* = 5.23 × 10^−3^, A: F(1, 85) = 32.3, *p* = 1.47 × 10^−6^, SxA: F(1, 85) = 3.21, *p* = 0.162). Similarly, the number of arm choices were both reduced after aripiprazole treatment (Fig. 4C; A: F(1, 85) = 114, *p* = 1.18 × 10^−15^). In the open field test, aripiprazole and spironolactone showed a significant statistical interaction, but no significant spironolactone effect, neither on aripiprazole nor on placebo-treated mice (Fig. 4D; SxA: F(1, 85) = 8.10, *p* = 0.0186; P-S: F(1, 38) = 2.59, *p* = 0.116; A-S: F(1,58) 3.97, *p* = 0.0511).

Interestingly, the circadian activity amplitude, measured with a score for nocturnality of activity, was significantly reduced after aripiprazole treatment (Fig. 4B; A: F(1,85) = 23.27, *p* = 3.49 × 10^−5^). When looking at the activity over the 24 h period, it becomes clear that the reduced amplitude results from a lower nighttime activity, while activity during the light phase was similar to the respective placebo controls (Fig. 4E, F).

### Dimensionality reduction analysis reveals interactions between aripiprazole and spironolactone treatment

To obtain an easily comprehensible overview of the changes in the neurocognitive and behavioral profile of *Tcf4*SD mice in response to the different treatments, we computed a canonical discriminant analysis (CDA). CDA is a supervised form of dimension reduction analysis^37^. The outputs of this analysis are latent variables, or canonical components, consisting of a linear combination of single variables, similar to principal component analysis (PCA). However, in a CDA, these linear combinations are optimized for between-group variation and the weights (canonical coefficients) of single variables in this combination provide information on the importance of the respective variable for group separation^37^.

**Fig. 5:**
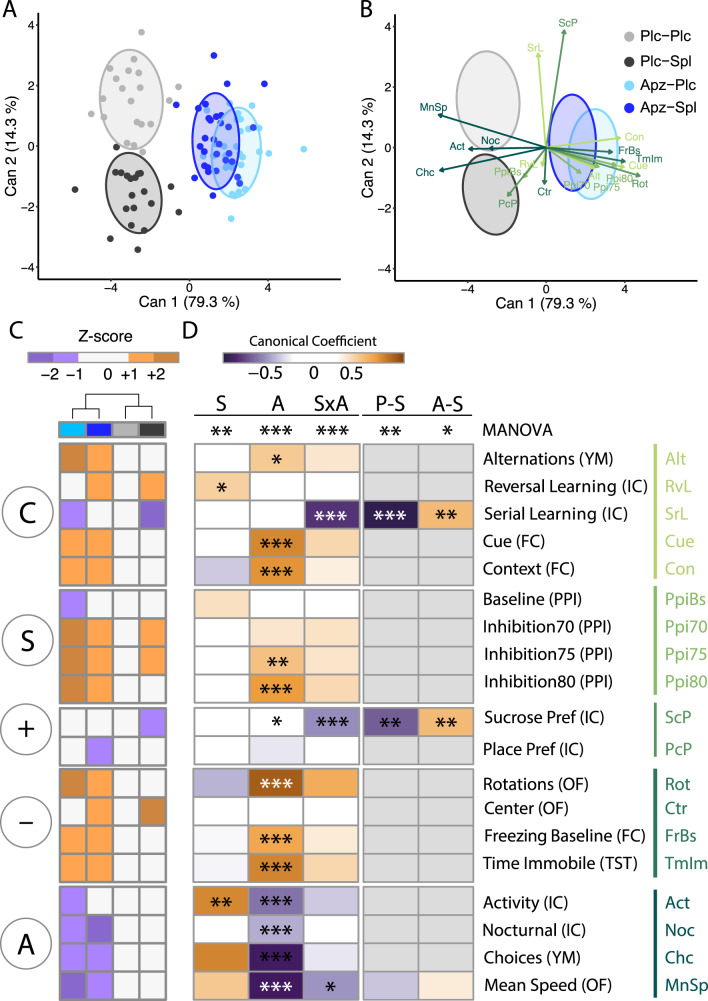
Canonical discriminant analysis reveals a clear interaction between aripiprazole and spironolactone treatment. **A** The dimension plot of the first two canonical components shows a clear separation of aripiprazole-treated groups from their respective placebo-treated references as well as the Plc-Spl from the Plc-Plc group. The first canonical component (Can1) explains 79.3% of the total canonical correlation, while the second component (Can2) only explains 14.3%. **B** The weights of the single variables (canonical coefficients) are plotted on top of the data ellipses, showing which variables drive group separation in this analysis. **C** The heatmap shows the difference of the mean Z-score of each treatment group compared to placebo control. The hierarchical clustering on top of the Z-score profiles supports the stronger and less specific effect of aripiprazole (blue colors) on the behavioral phenotype compared to spironolactone (dark shades). **D** In a multivariate ANOVA, we found an interaction of spironolactone and aripiprazole treatment (SxA: F(19,67) = 4.52, *p* = 2.09 × 10^−6^) with significant spironolactone effects in both placebo and aripiprazole-treated mice (P-S: F(19, 12) = 4.55, *p* = 5.07 × 10^−3^; A-S: F(19, 37) = 2.40, *p* = 0.0114). When looking at the CDA results from individual terms of the multivariate linear model and their corresponding simple-effects models, we find several strong contributions to the interaction of aripiprazole and spironolactone treatment. In contrast to the collapsed factor model shown in **A** and **B**, this analysis deconvolutes the two factors and their interaction strictly. The corresponding simple-effects models suggest that the direction of spironolactone’s effect depends on aripiprazole treatment, having positive weights in the placebo group’s canonical component and negative weights in the aripiprazole-treated groups. *p < 0.05, **p < 0.01, ***p < 0.001, n.s. not significant; *p*-values are FDR-adjusted and refer to Wilk’s lambda testing in a multivariate two-way ANOVA with subsequent univariate two-way ANOVAs; In case of a statistically significant interaction, the spironolactone main effect was tested for placebo and aripiprazole treatment in simple-effects ANOVAs; *n* = 19/19/30/30; Plc placebo, Spl spironolactone treatment, Apz aripiprazole treatment, S spironolactone term, A aripiprazole term, SxA interaction term, P-S spironolactone effect in placebo-treated mice, A-S spironolactone effect in aripiprazole-treated mice.

When looking at the four groups individually in a dimension plot of the first two canonical components, the Plc-Spl and Plc-Plc groups are clearly distinguishable from each other as well as from the aripiprazole-treated only mice (Apz-Plc) (Fig. 5A). The two aripiprazole groups, Apz-Plc and Apz-Spl, show a large overlap in this visualization of the complete phenotypic space. This suggests that the combined treatment of spironolactone and aripiprazole is less effective as spironolactone alone. This is further supported by the small portion of only 14.3% of the total canonical correlation that is explained by the second canonical component (Can2), which mostly separates the spironolactone-treated from the respective placebo groups. The first canonical component (Can1), which separates aripiprazole-treated from placebo mice, explained the majority (79.3%) of canonical correlation. In addition, in the data ellipse representation, the dark blue combined treatment Apz-Spl ellipse lies closer than the cyan aripiprazole-only Apz-Plc group to both gray no-aripiprazole Plc-Plc and Plc-Spl groups. This already suggests an interaction between spironolactone and aripiprazole treatment.

The canonical coefficients of single variables were next plotted as vectors with their base in the origin and with x and y coordinates of their tips depending on their weight in each canonical component (Fig. 5B). This visualizes the contribution of each variable to the structure of the canonical components. Can1 shows major influence of aripiprazole treatment on mean speed (MnSp), cued fear memory (Cue), number of choices (Chc), rotation rate (Rot), and prepulse inhibition (Ppi70, Ppi75, Ppi80), whereas Can2 appears to be dominated by sucrose preference (ScP), serial reversal learning performance (SrL) and place preference (PcP), with smaller contribution of first reversal (RvL), PPI baseline (PpiBs), center time and the number of choices (Chc). The vectors were colored according to their assigned RDoC domain. From this shading, it becomes clear that variables of the activity and regulatory domains (darkest shade) are strongly influenced by aripiprazole’s decrease in locomotor activity that strongly contributes to Can1.

Next, we visualized the neurocognitive and behavioral profiles of all treatment groups in a single heatmap of z-score transformed values for an intuitive and easy access to the results (Fig. 5C). Similar to the dimension plots (Fig. 5A, B), the unsupervised clustering of the treatment conditions separates the Plc-Plc and Plc-Spl groups from the aripiprazole groups (Apz-Plc; Apz-Spl) (Fig. 5C). As described previously^17^, not all visible differences are significant in statistical tests and, therefore, the combined CDA heatmap provided next to it with the corresponding ANOVA test results superimposed (Fig. 5D).

While in the CDA approach, shown in Fig. 5A, B, each of the first two canonical components can be mostly attributed to either factor due to the differences in their effect on the mice’s behavioral profile, this separation is not clear-cut and the canonical coefficient vector representations should be interpreted cautiously. However, the dataset can also be forwarded to the CDA as a bifactorial linear model, with one CDA result per term: spironolactone (S), aripiprazole (A), and their interaction term (SxA). This offers a clear separation of main effects and identifies potential dependencies between factors, similar to the corresponding ANOVA. The canonical coefficients of the first canonical component can then be visualized in a heatmap with the ANOVA results overlayed to give insight in the structure of each term’s effect (Fig. 5D).

Overall, spironolactone and aripiprazole treatment show a significant interaction in a multivariate ANOVA (Fig. 5D; SxA: *p* = 2.09 × 10^−6^). In the corresponding simple-effects ANOVAs, spironolactone was found to affect the behavioral profile of *Tcf4*SD mice, both with and without aripiprazole (P-S: *p* = 5.07 × 10^−3^, A-S: *p* = 0.0114). This suggests that aripiprazole co-treatment does not abolish but changes the effect of spironolactone. Looking at the corresponding univariate contrasts, there are three variables showing a significant interaction term: serial reversal learning (SrL; SxA: *p* = 1.80 × 10^−8^), sucrose preference (ScP; SxA: *p* = 1.34 × 10^−5^), and mean speed (MnSp; SxA *p* = 0.0186). Interestingly, in SrL, we found aripiprazole to interfere with spironolactone’s beneficial effect on learning performance, with co-treated mice still performing significantly better than placebo-treated controls (Figs. 2C, 5D; P-S: *p* = 5.39 × 10^−8^, A-S: *p* = 5.84 × 10^−3^). This mirrors the effect found in the multivariate ANOVA. In contrast, in the sucrose preference test, aripiprazole negated the spironolactone-induced reduction in sucrose preference in the combination treatment (Figs. 3A, 5D; P-S: *p* = 3.54 × 10^−3^, A-S: *p* = 1.61 × 10^−3^). For mean speed in the open field test, no significant simple effects were found (Figs. 4A, 5D; P-S: *p* = 0.116, A-S: *p* = 0.0511). In conclusion, the interactions are not consistent between the affected traits.

When looking at the RDoC domain level, the effects observed in the other cognitive systems domain variables are not consistent as well. However, the sensorimotor systems, the negative valence systems, and arousal/regulatory domains are mostly impacted by aripiprazole treatment. The only significant main effect found for spironolactone outside of the cognitive system is an increase in general activity in the IntelliCage (Fig. 4A; S: *p* = 0.0186). Notably, the structure of the S term’s canonical component puts high weights on choices and mean speed, although neither of them showed a significant difference in the corresponding univariate or simple-effects ANOVAs (Chc: S: *p* = 0.451; MnSp: P-S: *p* = 0.116, A-S: *p* = 0.0511). However, the mean number of choices in the Y-maze test is slightly higher for spironolactone-treated mice (46.6) compared to placebo (44.5), supporting this finding, and suggesting a subtle effect on novelty-induced activity.

## Discussion

In this study, we used the PsyCoP platform to validate the effect of spironolactone alone and in combination with the antipsychotic aripiprazole on cognition and behavior in the *Tcf4*SD 2-hit mouse model. We think that the PsyCoP workflow is well suited to guide phenotypic compound screens and preclinical drug development in areas of currently unmet therapeutic needs in the treatment of psychiatric disorders.

We found that spironolactone improves serial reversal learning and aripiprazole cue and contextual fear memory in *Tcf4*SD mice, but also that both drugs partially interfere with each other. For example, aripiprazole reduces the beneficial effect of spironolactone in the combination therapy. A similar interaction was detected for the modulation of novelty-induced activity. Moreover, we observe an increase in negative valence measures and activity-dampening effects upon aripiprazole treatment.

The presented translational compound validation study was inspired by an actual ongoing clinical study of spironolactone as an adjuvant to antipsychotic medication^34^. Similar to the clinical trial, we tested the compounds in *Tcf4*SD mice only i.e., the disease model without a ‘healthy control’ group. In our opinion, the advantages of limiting these experiments to compound effect and interaction detection outweigh the disadvantages. First, following the 3R principle, it should be one of the prime goals in designing animal studies to reduce the number of animals required as much as the aim, the methods, and the model system allow for. Furthermore, in the field of drug repurposing, the compounds in question have typically already been tested in wild-type mice before and further studies could still be performed in case of unexpected drug activities in the disease model. Moreover, in accordance with the clinical trial, we tested spironolactone not only in monotherapy, but also in combination in order to detect drug interactions, which would inflate animal numbers quickly if ‘healthy controls’ would be included from the start.

The most prominent finding of our study with the 2-hit *Tcf4*SD mouse model is the significant improvement of reversal learning by spironolactone alone and a substantial reduction of this effect by co-treatment of spironolactone and aripiprazole (see below). We also observed a slight, yet significant impairment in the first reversal test by spironolactone. Dichotomous effects on cognition have been observed also in humans in a combined psychosocial stress and acute spironolactone pre-treatment paradigm, where working memory was impaired, while long-term memory was improved^38^. Therefore, it might be possible that in the first reversal phase hippocampus-based memory retrieval may be dominating, whereas in the following serial reversal learning phases prefrontal cortex/striatum-based re-learning and/or procedural adaptation learning overrides the initial memory. As in a previous study^38^, different cognitive systems may be differentially sensitive to psychosocial stress and spironolactone treatment.

Drug interactions are very common and constitute a major issue in clinical practice as an average patient with a psychotic disorder usually receives a combination treatment. We show here that a spironolactone and aripiprazole combination appears to be detrimental rather than beneficial for rescuing learning performance. This effect might be specific to the co-treatment of spironolactone with aripiprazole and absent or less pronounced for other antipsychotic drugs. A cue to this specificity is the fact that spironolactone is thought to act by inhibiting NRG1-mediated hyperphosphorylation of the ErbB4-receptor in the cortex^23^, while aripiprazole was found to reduce NRG1 and ErbB4 protein levels in several schizophrenia-relevant brain regions in rats^39^. Therefore, the interference of both drugs could be due to a convergence on the NRG1-ErbB4 pathway. This hypothesis could be tested in the future with additional antipsychotics that are commonly applied in the clinic such as quetiapine, risperidone, and even clozapine. Thereby, patient selection for future clinical trials could be improved accordingly.

The antipsychotic chosen for this study, aripiprazole, is already known to have various effects on behavior in humans and mice. Notably, cognitive improvement after chronic, and sometimes even acute treatment, has been found in other rodent models before: Memory function in the Morris water maze^40^, slight improvement of working memory in the Y-maze test^41^, as well as short- and long-term memory retention in aversive learning following acute intraperitoneal injection^42^. We could reproduce some of these findings in our model, supporting PsyCoP’s potential as a standardized profiling tool. Specifically, we did find improvement of spatial learning flexibility and memory performance after fear conditioning.

However, for the interpretation of the latter effects, it has to be taken into account that aripiprazole has a considerable effect on locomotor activity, although this side effect is considered to be modest compared to other antipsychotics^43,44^. Aripiprazole was shown to prevent amphetamine-induced motor hyperactivity in rats^45^ and mice^46^. An activity-dampening effect was also found in mice without stimulating pre-treatment^42^. Whether our results indicate the amelioration of hyperactivity found in *Tcf4*SD mice^17,18^ or rather a sedative effect due to high dosage cannot be fully resolved without further studies. We have also validated aripiprazole’s beneficial effect on PPI, which was shown in healthy humans with low-gating properties^47^, as well as in mice receiving PPI-disrupting treatment in form of the NMDA receptor antagonists phencyclidine^48^ and MK-801^49^.

With the help of PsyCoP, we were able to characterize the behavioral effect profile of our compound of interest, spironolactone. So far, studies in healthy humans with spironolactone revealed mixed results on cognition, likely depending on age and disease context^50^. However, most human studies have been focused on affective disorders, such as major depression and borderline personality disorder, given the primary mode-of-action of spironolactone as a mineralocorticoid receptor antagonist and its impact on stress hormones^50^. For schizophrenia, there is no study published that focuses on cognition, although one study shows that spironolactone, as add-on to risperidone, may improve positive and negative symptoms^51^. In animal models, spironolactone was previously shown to improve cognitive performance, e.g., rescuing working memory performance in *Nrg1*tg mice^23^. In addition, a study in a female diabetes mellitus type 2 mouse model demonstrated improvement of spatial memory in response to spironolactone treatment in both diseased and healthy mice^52^. In an Alzheimer’s disease model, spironolactone prevented cognitive decline, thought to be meditated via increasing BDNF protein levels^53^ and schizophrenic patients have been shown in a meta-analysis to have reduced plasma levels of this neurothrophin^54^. Here, we validated a beneficial effect on reversal learning, but also analyzed the “side effect profile” of spironolactone, detecting an effect on general locomotor activity. We observed trends in a similar direction in novel environments such as in the open field and the Y-maze tests. Notably, hyperactivity is typically used as an indicator of dopaminergic actions related to positive symptoms in mouse models of mania and psychosis^55–57^. This effect might have therefore been overlooked without a full PsyCoP profiling.

Based on these findings, we speculate that a beneficial effect of spironolactone on flexibility learning will be dampened, at least in aripiprazole-treated patients in the ongoing clinical trial. This is in line with the hypotheses that antipsychotics with dopaminergic partial agonism, such as aripiprazole, may have a procognitive effect in psychotic disorders^58^. Of note, in another recent clinical trial, which focused on effects on positive and negative symptoms of schizophrenia, risperidone-treated patients benefitted from spironolactone add-on^51^. Thus, spironolactone is a promising target for drug repurposing efforts and combination therapy of schizophrenic patients, as it was shown to have ameliorating effects on cognitive dysfunction in several mouse models so far. We are aware of the fact that preclinical trials in rodent models often have limited predictive translational value^59^. Nonetheless, our study may at least be helpful in re-designing or stratifying clinical studies, specifically in the choice of the antipsychotic drug to be combined (or better not) with a repurposing candidate.

In our opinion, it is crucial for translational psychiatry to test multiple mouse models with a variety of genetic and environmental influences for compound testing. The RDoC concept provides a suitable framework for informing this and future translational studies. RDoC or any other classification system for behavioral-endophenotypes in combination with a standardized platform like PsyCoP may allow for more powerful types of guiding analyses such as side by side comparisons of neurocognitive profiles from different mouse models. This may allow researchers to find appropriate models for specific research questions, not on the level human disorders, but on neurobiological systems that are possibly associated with specific endophenotypes of psychiatric disorders.

Furthermore, making this kind of data publicly available and collecting it in appropriate databases such as the Mouse Phenome Database^60^ will enable future meta-analyses and will increase the value of translational psychiatric research substantially.

## Materials and methods

### Animals and husbandry

The C57Bl/6N mice used for backcrossing were obtained from Charles River Laboratories GmbH, Sulzfeld, Germany.

Mice were provided with chow and water ad libitum. Access to water was temporarily restricted in the IntelliCage system for place learning. *Tcf4* transgenic (*Tcf4*tg) mice were littermates and weaned after three weeks. Only male mice were used due to the sex-specific restriction of social defeat. The mice were kept in type IV cages (Tecniplast 2000, 612 × 435 × 216 mm, 2065 cm²) in groups of litters between 8 and 15 mice. Experiments were conducted with mice 9–14 weeks of age.

### Drug treatment

Treatments were chosen to resemble the currently running clinical trial, in which patients with a stable antipsychotic treatment and no more than two antipsychotics, but not clozapine, were included^34^. Arguably, this approach is close to actual clinical applications, where most patients are continuously treated with antipsychotics to ameliorate positive symptoms and it is for ethical reasons not possible to withdraw patients from treatments for a new trial. In this study, we chose the second-generation antipsychotic aripiprazole, because the antipsychotic agent should not be eliminated primarily renally to avoid interference with spironolactone treatment via this route.

All mice were treated orally via the chow to minimize handling and stress before and during experiments. Chronic oral drug treatments began two weeks before the behavioral test battery at seven weeks of age. Micronized spironolactone was administered at 75 mg/kg/day, based on our previous work^23^, a high oral bioavailability, a tolerated human dose of 400 mg, and the conversion to a mouse equivalent dose^61^. The diet was custom-made and gamma-irradiated (Ssniff, Germany), assuming a daily chow consumption of 150 g/kg/day^62^. Aripiprazole was administered with a target dosage of 3 mg/kg/day. The aripiprazole dose was based on the very limited set of pharmacokinetic data in mice, studies with chronic administration^63–66^, human to mouse dose conversion^61^, and the requirement for unimpaired motor function and vigilance.

### Behavioral procedures

Experimental mice were handled with clear polycarbonate tubes, which has been shown to reduce stress in laboratory mice^67^. Mouse studies were conducted in accordance with the German Animal Protection Law. All experiments were conducted at approximately the same daytime during the light phase. Experiments in the IntelliCage system were monitored continuously. Before each of the daytime experiments, the home cage was placed in the experiment room for 10 min before the first test for acclimatization. Test equipment was first wiped with SDS solution and then with ethanol to remove remaining olfactory cues. This cleaning routine was done before and after each trial if not stated otherwise. The animals went through all the procedures in the order listed (Fig. 1A). The procedures were essentially identical to those described in detail in the supplementary methods section of Volkmann et al.^17^. Short summaries for providing the information necessary to understand the contents of this article as well as differences in the tests and the data analysis are provided below.

### Social defeat

The resident intruder social defeat paradigm of psychosocial stress was essentially performed as described in Brzózka et al.^68^, starting at an age of 4 weeks. Old FVB/N male mice kept in isolation were used as resident stressor mice. On each of 21 consecutive days, the test mice (intruders) were transferred to a resident’s home cage individually. After the first physical attack, the intruder was isolated from the resident by a metal mesh cage (75 mm × 115 mm × 60 mm) for another 30 min, allowing for olfactory, auditory, and visual contact only. The test mice were then marked and returned to their home cage. The time of day for the pairing was randomized and always started in the light period. The order pairing was regularly rotated between cages. Intruders and residents were matched according to a rotating scheme optimized to avoid repeated contacts, which could lead to mutual familiarization.

### Transponder implantation

For identification of the test mice in the IntelliCage system, an RFID transponder was implanted in the neck of all test mice. For pain management, 200 mg/kg metamizol solution was administered p.o. from a syringe before and carprofen 5 mg/kg s.c. during surgery. Isoflurane was used as anesthetic. Eyes were protected with Bepanthen eye ointment. The skin was then shaved in the neck region, disinfected with 70% alcohol, and the transponder (1.4 × 11 mm) was placed under the skin using an injector. The wound was closed with one or two stitches and a drop of tissue glue to protect against a loss of the implant. After the surgery, the mice had at least 6 days of recovery before the first test.

### Open field test

The open field test was performed as described previously^17^. Mice were tested starting 2 h after the start of the light cycle in a box-shaped white open field arena (50 × 50 × 50 cm). Mice were placed in the box with their nose in direction to the wall and monitored for 10 min. Illumination was kept at about 1600 lux. Data was acquired using ANY-maze (Stoelting, Wood Dale, IL, USA). The center area was defined as the total area excluding a 5 cm periphery strip and 10 cm corner squares.

### Y-maze test

The Y-maze test was performed as described in the earlier PsyCoP study^69^. In brief, test mice were placed in a Y-shaped arena with identical arms (A, B, C) with their noses in direction of the center area. They were monitored for 10 min at 30–50 lux. ANY-maze (Stoelting, Wood Dale, IL, USA) was used for recording. Spontaneous alternations were counted as full sequences without repetition (e.g., A-B-C).

### IntelliCage system

The IntelliCage system (www.tse-systems.com/product-details/intellicage) is a commercial fully automated conditioning device, which is placed in a type IV cage. The device consists of four corners, each with two water bottles, one on each side of the corner. These water bottles can be accessed via doors. The conditions under which any of these doors open is freely programmable. In our learning experiments, test mice had to poke at a door in an individually assigned ‘correct’ corner in order to open it and to access the water bottle. Three types of events were recorded during IntelliCage experiments: visits to a corner, nose pokes, and licks at water bottles. Mice were kept in their established social group when transferred to their IntelliCage. The mice were transferred with some of their used bedding in order to reduce aggressive behavior^70^.

The experiments were switched at the same daytime during the light phase at their lowest activity. The IntelliCage tests consisted of the following stages:

1. Five days of acclimatization and activity monitoring.
2. Two days of place preference, where each mouse could open the doors of only one of the four corners with a nosepoke.
3. Five days of serial reversal learning. Individual correct corners were switched daily in a pseudo-randomized order.
4. One day of sucrose preference test, where mice could freely access all bottles. All left bottles were filled 4% sucrose solution, all right bottles with plain water.

Success rate in preference paradigms was measured using the preference score (A − B)/(A + B) with A being the correct trials (visits in the assigned corner with at least one nosepoke) or the number of licks at a sucrose solution bottle and B being incorrect trials (visits with nosepoke in non-assigned corner) or licks at a bottle containing plain water. For place preference, the score was weighted to compensate for random chance. Flexibility of learning was tested in the first reversal phase of the serial reversal learning experiment as the number of trials needed to reach the learning criterion in a sequential probability ratio test (SPRT) as a measure of learning speed. The strategy-finding performance was measured as approximate area under the learning curve across all reversal phases as the sum of the rolling mean between two neighboring phases, derived from the SPRT criterion in each phase. In addition, we analyzed the success rate (successes/trials) in each learning phase and its mean, as well as the rate of rewarded trials (trials with licks/total trials).

### Pre-pulse inhibition test

Pre-pulse inhibition was performed as described in our PsyCoP study^17^. Before the actual test, all mice were habituated to the enclosure for 10 min for 3 days, providing lighting and white background noise at 65 dBA. Startle response was measured using SR-LAB (San Diego Instruments, San Diego, USA). After ten short-term habituation trials at the main pulse sound level (115 dBA, based on a previously acquired input/output curve), pre-pulse inhibition was tested at 5 dBA, 10 dBA, and 15 dBA above background with 10 trials each. Trials were pseudo-randomized with inter-trial intervals between 8 and 22 s.

### Tail suspension test

The tail suspension test was performed as described in the PsyCoP study^69^. In summary, mice were suspended, hanging by their tails about 30 cm above ground for 6 min. Lighting was kept at 1600 lux during the test. ANY-maze (Stoelting, Wood Dale, IL, USA) was used for recording and immobility detection. Forelimb movement alone was not counted as mobile.

### Fear conditioning test

Fear conditioning was performed as described earlier^71^. A commercially available fear conditioning setup from Ugo Basile (Siena, Italy) was used. For conditioning, a black and white striped background, white noise, the grid floor, and ethanol smell were defined as context. After recording baseline freezing and habituation to the context, a 30 s tone was played as the cue. After the tone, a 0.6 mA foot shock was delivered through the grid floor. The conditioning block was repeated once without the habituation phase. After 24 h, the mice were placed in the same context for 2 min and monitored for freezing behavior. After another 24 h, the mice were placed in a clear plastic cylinder of roughly the same size as the conditioning box on a rough gray floor, and no ethanol use between trials. After 2 min habituation, the cue was played and the animals were monitored for freezing for another 2 min. Freezing behavior was quantified using ANY-maze (Stoelting, Wood Dale, IL, USA).

### Statistical analysis

The software used as a graphical user interface for data processing was FlowR (XBehavior, Dägerlen, Switzerland). The CDA and plotting routine was done using custom scripts complementing the FlowR bundle using the RStudio IDE (RStudio, Boston, MA, USA).

For box and whisker plots, we used the R package *ggplot2*^72^. Single data points were plotted next to the corresponding box. Tukey’s method was used for whiskers. *P*-values were derived from univariate two-way ANOVA with type 2 sum of squares and subsequent false discovery rate (FDR) adjustment to an FDR of 0.1. The multivariate linear model used for the statistical test was also used in a multivariate ANOVA from a Wilk’s lambda distribution.

For tests with a significant effect in the interaction term, a simple-effects ANOVA was calculated and indicated for each genotype level.

For heatmaps, the *pheatmap* package was used. Variables were organized in blocks in accordance with their preassigned RDoC domain^21^.

The dimension reduction procedure was performed as follows: first, missing values in the data matrix were filled with estimators generated by the non-linear iterative partial least squares (NIPALS) algorithm as implemented in the function *nipals* in the *ade4* R package. The resulting reconstituted matrix was forwarded to the *candisc* function in the *candisc* R package, which computes a canonical discriminant analysis (CDA). CDA finds linear combinations of variables (canonical components) with optimal canonical correlations within groups, giving improved group separation over more commonly used dimension reduction methods like principal component analysis (PCA).

From CDA, we generated two plots: a dimension plot with data ellipses covering 75% of all data points in each group and a heatmap showing the weights (canonical coefficients) of each single variable in the canonical component, optimized for each term of the underlying linear model: spironolactone treatment, aripiprazole treatment, and their interaction. In addition, the same analyses were run for the simple-effect models of each aripiprazole treatment level. This gives insights in the importance of each variable for separation of the samples along each factor and their interaction.

For the Z-score heatmap, the raw data table was Z-transformed, and the group means were computed. These were centered on the placebo control group in order to reflect compound effects on that reference. The group mean Z-scores were plotted and clustered using the *pheatmap* package. Hierarchical clustering was done using Manhattan distances.

## Supplementary information

Supplemental Figure 1

Supplemental Figure 2

Supplemental Figure 3

Supplemental Table 1

Supplemental Table 2

Supplemental Table 3

## Data Availability

The code written for this study can be found on GitHub (https://github.com/volkmannp/PsyCoP) and is provided with an example dataset. All scripts are available as standalone R scripts and as a FlowR bundle. The complete set of raw and processed data can be found in Suppl. Table 2.
