# Supplementary material for: Spironolactone alleviates schizophrenia-related reversal learning in Tcf4 transgenic mice subjected to social defeat
Source: Schizophrenia (Heidelb). 2022 Sep 29;8(1):77. doi: 10.1038/s41537-022-00290-4 (PMC9519974; doi:10.1038/s41537-022-00290-4)
Supplement: Supplementary file 1 — Supplemental Figure 1 [file 41537_2022_290_MOESM1_ESM.pdf]

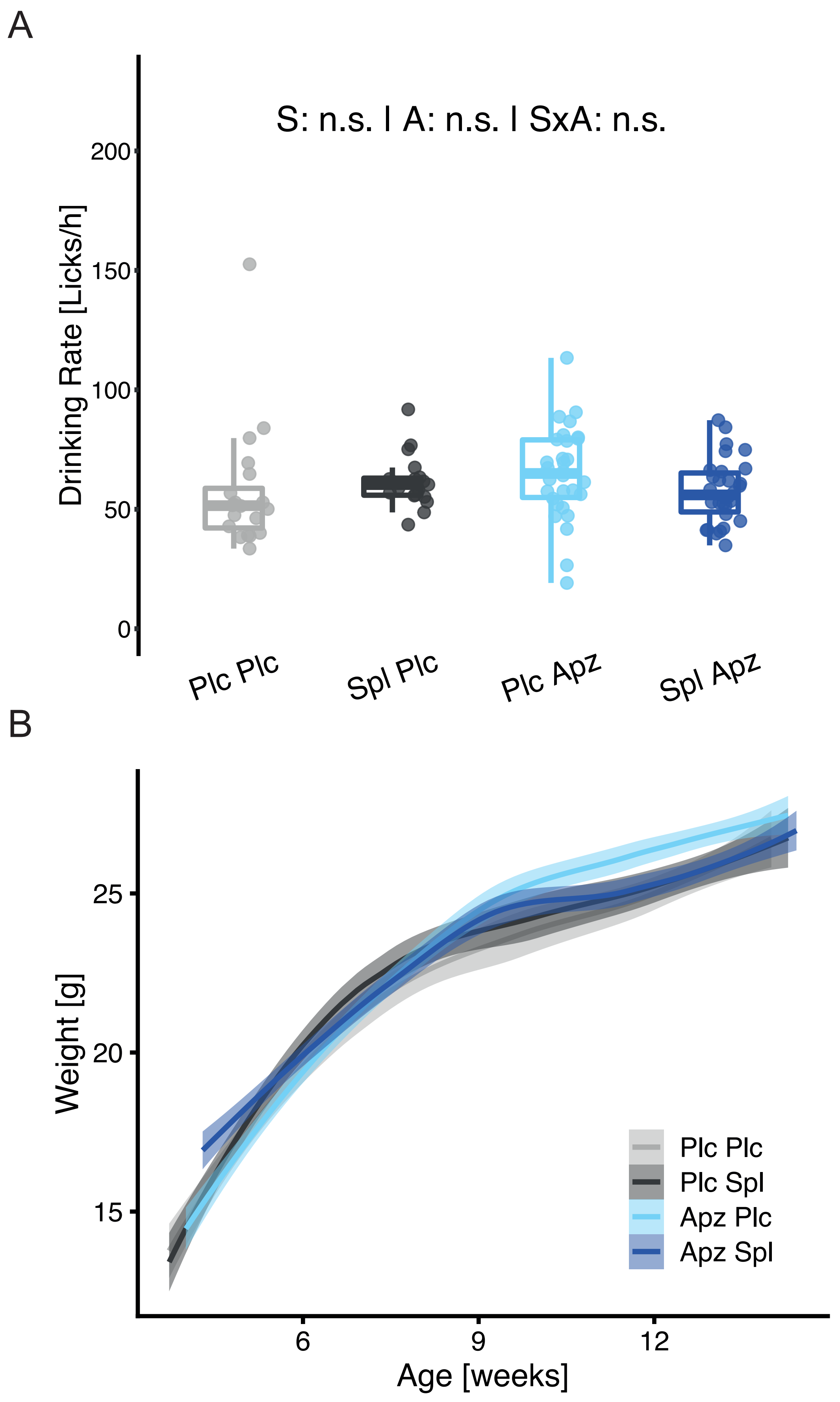

Suppl. Figure 1. Control of drinking behavior and weight of test mice. (A) The drinking rate was documented during all IntelliCage-based tests. During this period no significant differences in in water consumption could be detected, indicating that spironolactone treatment did not lead to an increase in dosage due to diuretic effects. (S: $F(1, 95) = 0.588$, $p = 0.4450$; A: $F(1, 95) = 0.280$, $p = 0.5981$; SxA: $F(1, 95) = 2.48$, $p = 0.1184$) (B) Moreover, the mean weight curves do not show a persistent weight loss in consequence of neither spironolactone nor aripiprazole treatment. The line depicts the mean, while the ribbon visualizes the standard error. n.s. not significant; p-values refer to a Type II sum of square univariate two-way ANOVA Plc = Placebo/no treatment; Spl = Spironolactone treatment, Apz = Aripiprazole treatment; S = Spironolactone term; A = Aripiprazole term; SxA = Interaction term
