# Supplementary material for: Spironolactone alleviates schizophrenia-related reversal learning in Tcf4 transgenic mice subjected to social defeat
Source: Schizophrenia (Heidelb). 2022 Sep 29;8(1):77. doi: 10.1038/s41537-022-00290-4 (PMC9519974; doi:10.1038/s41537-022-00290-4)
Supplement: Supplementary file 2 — Supplemental Figure 2 [file 41537_2022_290_MOESM2_ESM.pdf]

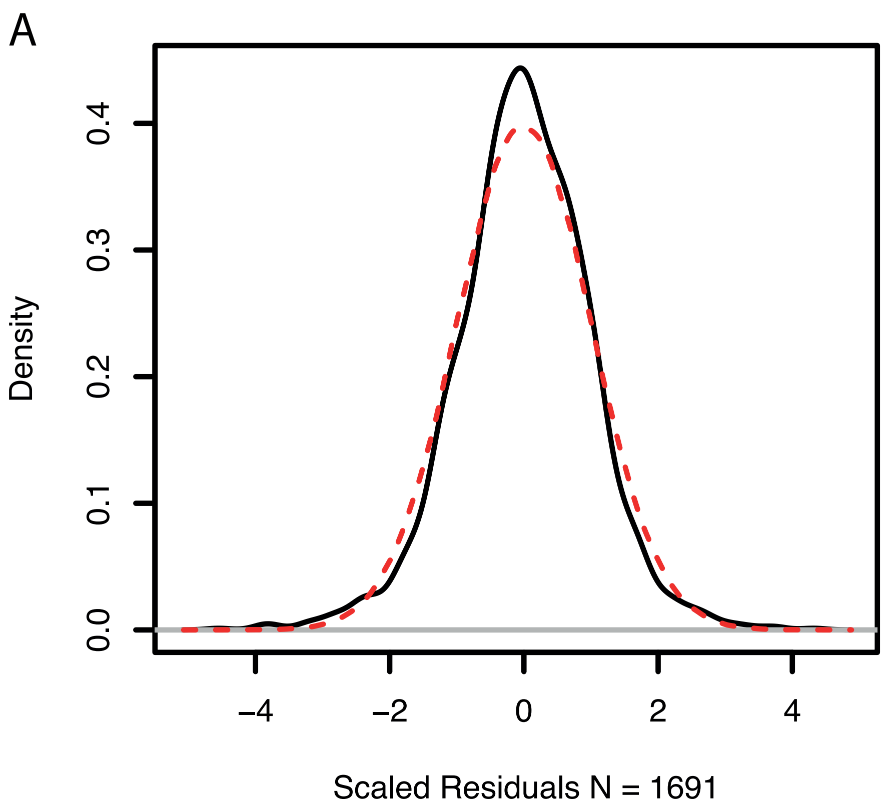

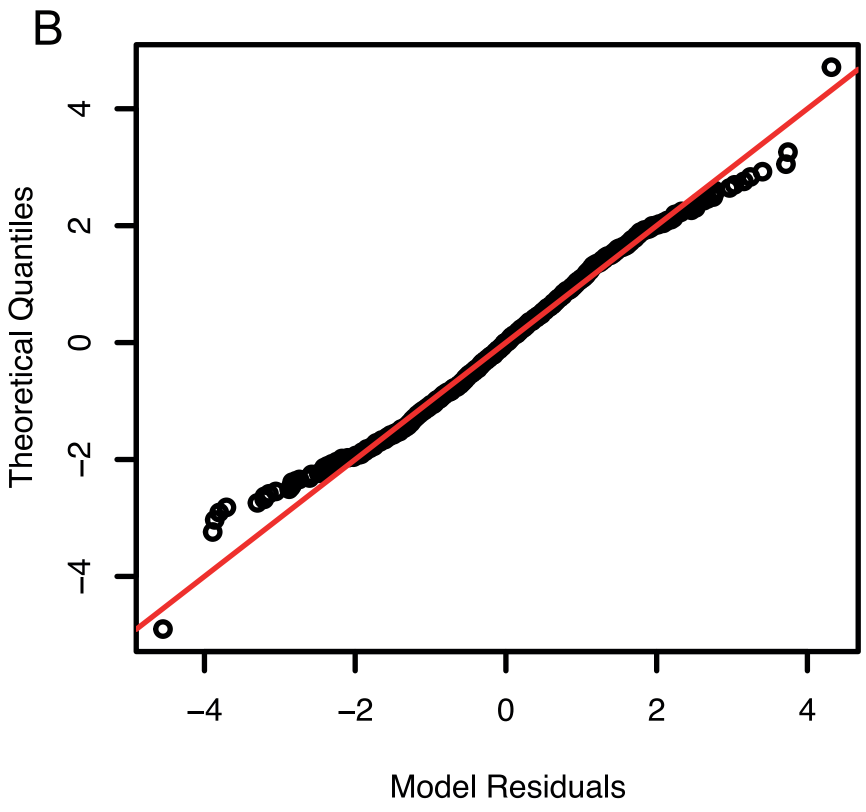

Suppl. Figure 2. Visual controls confirm approximate normal distribution of the multivariate linear model's residuals. Analysis of variance (ANOVA) type of tests assume a normal distribution of the residuals tested. (A) Shows a density plot of the scaled model residuals (black line) of the fitted multivariate linear model with a superimposed normal distribution (red dashed line). (B) displays a quantile-quantile (QQ) plot comparing the distribution of our model residuals with a normal distribution. The red line indicates identity of the distributions. Despite minor deviations from normality being visible, the overall assumption of an approximate normal distribution is not violated.
