# Supplementary material for: Spironolactone alleviates schizophrenia-related reversal learning in Tcf4 transgenic mice subjected to social defeat
Source: Schizophrenia (Heidelb). 2022 Sep 29;8(1):77. doi: 10.1038/s41537-022-00290-4 (PMC9519974; doi:10.1038/s41537-022-00290-4)
Supplement: Supplementary file 3 — Supplemental Figure 3 [file 41537_2022_290_MOESM3_ESM.pdf]

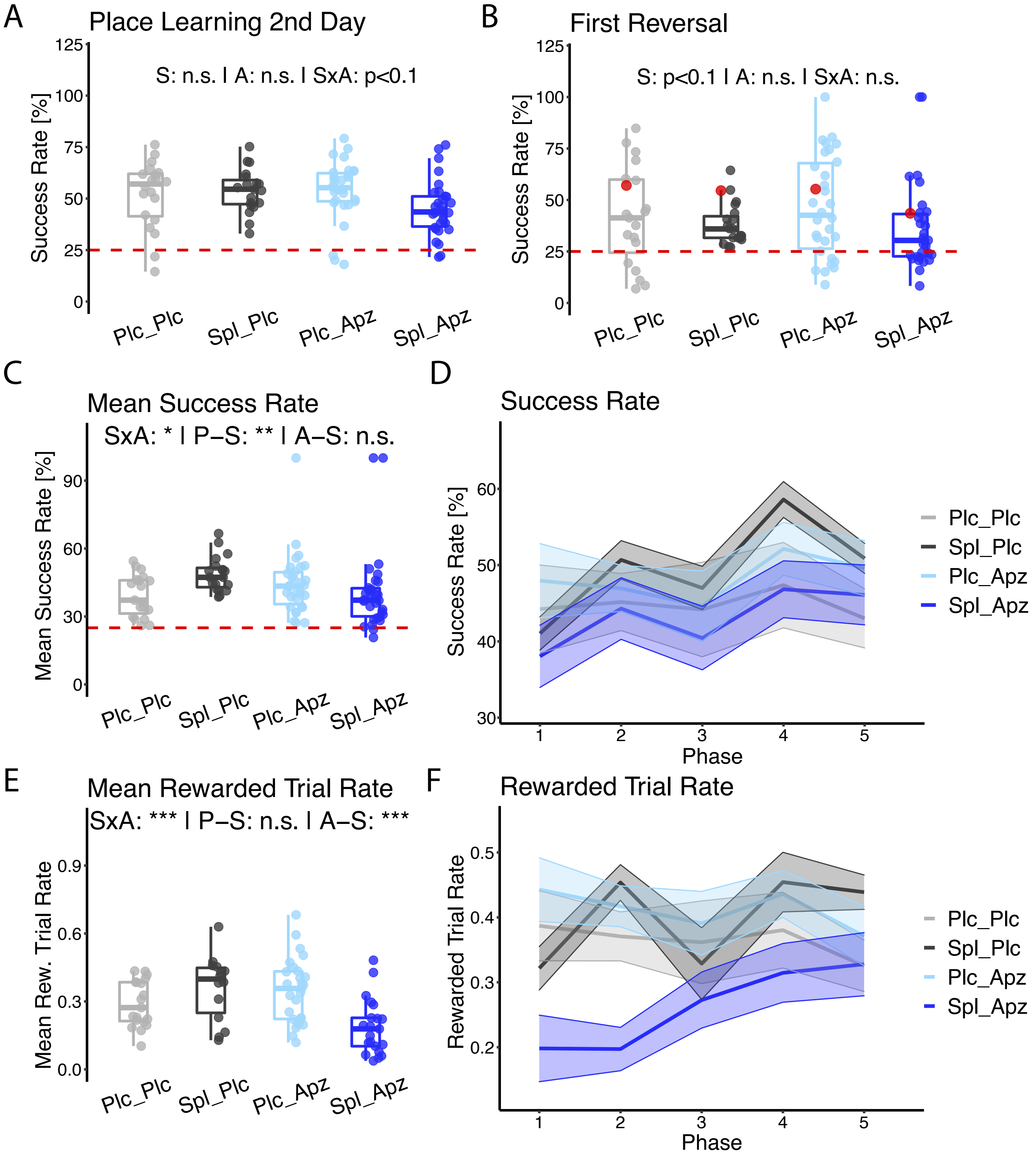


**Suppl. Figure 3. Extended data on the serial reversal learning task.** (A) Success rate in the last part of place learning with random chance visualized as red dashed line. (B) Success rate in the first reversal phase and median success rate of the last part of place learning (red dot) as plotted in (A) to visualize the drop in all four groups. (C) Mean success rate across all reversal phases. The red dashed line indicates random chance. (D) The mean success rate plotted against the individual phases. (E) The overall means of the rewarded trial rates (rate of trials with licks). (F) The mean rewarded trial rates for each phase. Ribbons in line graphs represent the standard error of the mean (SEM). Data shown in box plots were plotted with whiskers extending to no more than 1.5-fold IQR; * $p < 0.05$, ** $p < 0.01$, *** $p < 0.001$, n.s. not significant; p-values are FDR-adjusted and refer to Wilk's lambda testing two-way ANOVA; n = 19/19/30/30; Plc = placebo, Spl = spironolactone treatment, Apz = aripiprazole treatment; S = spironolactone term, A = aripiprazole term, SxA = interaction term, P-S = spironolactone effect in placebo-treated mice, A-S = spironolactone effect in aripiprazole-treated mice.
